# Compressed variance component mixed model reveals epistasis associated with flowering in *Arabidopsis*

**DOI:** 10.3389/fpls.2023.1283642

**Published:** 2024-01-08

**Authors:** Le Han, Bolin Shen, Xinyi Wu, Jin Zhang, Yang-Jun Wen

**Affiliations:** ^1^ College of Science, Nanjing Agricultural University, Nanjing, China; ^2^ State Key Laboratory of Crop Genetics and Germplasm Enhancement and Utilization, Nanjing Agricultural University, Nanjing, China

**Keywords:** epistasis, GWAS, 3VmrMLM, *Arabidopsis thaliana*, flowering-related traits

## Abstract

**Introduction:**

Epistasis is currently a topic of great interest in molecular and quantitative genetics. *Arabidopsis thaliana*, as a model organism, plays a crucial role in studying the fundamental biology of diverse plant species. However, there have been limited reports about identification of epistasis related to flowering in genome-wide association studies (GWAS). Therefore, it is of utmost importance to conduct epistasis in *Arabidopsis*.

**Method:**

In this study, we employed *Levene’s* test and compressed variance component mixed model in GWAS to detect quantitative trait nucleotides (QTNs) and QTN-by-QTN interactions (QQIs) for 11 flowering-related traits of 199 *Arabidopsis* accessions with 216,130 markers.

**Results:**

Our analysis detected 89 QTNs and 130 pairs of QQIs. Around these loci, 34 known genes previously reported in *Arabidopsis* were confirmed to be associated with flowering-related traits, such as *SPA4*, which is involved in regulating photoperiodic flowering, and interacts with *PAP1* and *PAP2*, affecting growth of *Arabidopsis* under light conditions. Then, we observed significant and differential expression of 35 genes in response to variations in temperature, photoperiod, and vernalization treatments out of unreported genes. Functional enrichment analysis revealed that 26 of these genes were associated with various biological processes. Finally, the haplotype and phenotypic difference analysis revealed 20 candidate genes exhibiting significant phenotypic variations across gene haplotypes, of which the candidate genes *AT1G12990* and *AT1G09950* around QQIs might have interaction effect to flowering time regulation in *Arabidopsis*.

**Discussion:**

These findings may offer valuable insights for the identification and exploration of genes and gene-by-gene interactions associated with flowering-related traits in *Arabidopsis*, that may even provide valuable reference and guidance for the research of epistasis in other species.

## Introduction

*Arabidopsis thaliana*, an important flowering plant, has emerged as a model organism for molecular plant genetics research in recent years (Koornneef and Meinke, 2010). Its compact genome, short life cycle, ease of cultivation, and abundant genetic resources make it widely utilized in fundamental biology, crop enhancement, and biotechnology. The flowering phase of *Arabidopsis* plays a crucial role in determining the precise timing of reproduction, seed, and fruit development. Therefore, studying the regulation and molecular mechanisms of flowering time in *Arabidopsis* remains an important area of research. By discovering the genetic factors and regulatory pathways affecting flowering time in *Arabidopsis*, it is possible to identify homologous genes and manipulate their expression in agronomic crops, optimize crop flowering time to adapt to specific environments and agricultural practices, improve crop yields, and produce crops that are more adapted to climate change and stress resistance.

Flowering in *Arabidopsis* has complex regulatory mechanisms and pathways, and the phenotypic material of flowering under different regulatory pathways is particularly important to elucidate the genetic mechanism of flowering (Qi et al., 2018). In the photoperiodic pathway, *Arabidopsis* perceives light signals through photoreceptors and transmits them to its biological clock. The biological clock, responsive to changes in day length, ultimately transforms the light signals into flowering signals via the CONSTANS (CO) gene (Imaizumi and Kay, 2006). Under long-day treatments, the CO gene facilitates flowering, whereas under short-day treatments, it retards the process (Teper-Bamnolker and Samach, 2005; Balasubramanian et al., 2006). In addition, vernalization plays a vital role in regulating flowering. By suppressing the activity of the FLOWERING LOCUS C protein, low-temperature induction during vernalization unlocks *Arabidopsis*’s flowering potential (Helliwell et al., 2015). In additional to the vernalization pathway, it was shown that the flowering time of *Arabidopsis* in 25-27°C short days was similar that in 23°C long days, suggesting that higher temperature promotes flowering in *Arabidopsis* (Balasubramanian et al., 2006). These studies indicate that in the research on flowering-related traits of *Arabidopsis*, factors such as photoperiod, vernalization, and temperature need to be considered.

Epistasis, referred to as loci-locus interactions (He et al., 2019), plays an important role in phenotypic variation and has received much attention over the years. As a major factor in molecular evolution (Breen et al., 2012), epistasis plays a crucial role in quantitative genetic analysis and is now one of the main causes of ‘missing heritability’ (Mackay and Moore, 2014; Upton et al., 2016). In *Arabidopsis*, flowering time as a complex quantitative trait is regulated by genes such as photoperiod, but also by other physiological processes such as temperature signaling and vernalization, which are both independent and interrelated. Therefore, these physiological processes involve a large number of loci and even genes that often interact with each other, and individual genetic loci or genes may have a small effect on flowering time in *Arabidopsis*, but together with other genes may have a large effect on phenotypic variation (Zhang et al., 2014), making it particularly important to investigate epistatic loci for flowering-related traits in *Arabidopsis*.

Recently, researchers have proposed many epistasis detection algorithms for complex traits based on traditional genome-wide association studies (GWAS) or artificial intelligence (AI). The most basic approach to explore epistasis is regression-based methods such as PLINK (Purcell et al., 2007), which has the advantage of high computational efficiency, rapid analysis of tens of thousands of markers and epistasis, and wide application in case-control datasets, but a high false positive rate. BOOST (Wan et al., 2010), which uses a Boolean representation of genotype data, can save memory space and improve computational speed at the same time, but it can only handle binary phenotype data and not for continuous quantitative traits such as yield and flowering time, which is a very limited application scenario. For continuous traits in plants, mixed linear model (MLM)-based methods perform better due to accounting for environmental factors, controlling for population stratification, and explaining cryptic correlations among individuals. QTXNetwork is a multi-locus mixed model proposed by Zhang et al. (2015). This method first detects each marker to identify potential quantitative trait nucleotides (QTNs), QTN-by-environment interactions (QEIs), and all the pairs of markers to identify potential QTN-by-QTN interactions (QQIs), and then all the potential QTNs, QEIs, and QQIs are placed into a genetic model to identify significant loci. However, the associated polygenic backgrounds in the first step were not taken into account. Ning et al. (2018) proposed a rapid epistatic mixed-model association analysis (REMMA) algorithm, which used the best linear unbiased prediction (BLUP) to predict additive and dominant effects, their epistatic effects and their variances, and then Wald Chi-squared test was used to identify the significance of all the effects. However, their power could be further improved. Multifactor dimensionality reduction (MDR) (Moore, 2004), a classical nonparametric machine learning method, was originally designed for identifying epistasis in case-control studies. Quantitative MDR (QMDR) (Gui et al., 2013; Yu et al., 2015) represents a robust, model-free extension of MDR accommodated for quantitative phenotypes. None of them, however, effectively address the challenges posed by limited interpretability and overfitting in AI and lengthy computation times required for genome-wide markers.

To overcome the above issues, Li et al (2022a; 2022b). established a compressed variance component mixed model method, named 3VmrMLM, to detect QTNs, QEIs, and QQIs while controlling for all the possible polygenic backgrounds. It reveals epistatic effects by reducing the number of variance components, while ensuring high statistical power. Additionally, the method efficiently reduces computation time and effectively addresses potential confounding factors arising from various polygenic backgrounds.

A number of gene-by-gene interactions associated with flowering time have been identified in *Arabidopsis*. For example, Zhao et al. (2022) identified a novel flowering repressor, *UBA2c*, and showed that the expression of a key flowering repressor gene, *FLM*, is promoted by inhibiting the histone modification *H3K27me3*, thereby suppressing premature flowering in plants. Hanano and Goto (2011) found that the interaction of *FD* with *TFL1* by BiFC assay induces *Arabidopsis* flowering repressor genes to fine-tune flowering time and inflorescence meristem tissue development, which in turn affects flowering time. However, most gene-by-gene interactions related flowering in *Arabidopsis* have been obtained by biological methods such as transcriptome analysis, and few gene-by-gene interactions have been identified by GWAS.

In this study, QQIs and QTNs for eleven flowering-related traits in natural populations of *Arabidopsis* were investigated using 3VmrMLM with data from https://www.Arabidopsis.org. Differentially expressed genes were identified under temperature, photoperiod, and vernalization treatments. Candidate genes and gene-by-gene interactions were identified by functional enrichment, haplotype and phenotypic difference analysis. Epistasis for flowering-related traits of *Arabidopsis* will help identify interacting genes and provide references for studying epistasis in other crops.

## Materials and methods

### Genotypic and phenotypic data

The dataset of *Arabidopsis* (Atwell et al., 2010) including the phenotypic and genotypic data were obtained from https://www.Arabidopsis.org. The dataset consisted 23 flowering-related traits, 199 individuals, and 216,130 markers.

Among 23 traits, we focused on eleven traits related to flowering under three different environmental conditions, including temperature, photoperiod, and vernalization treatments. They were Days to flowering time under Long Day (LD), Days to flowering time under Long Day with vernalization at 4°C during 5 weeks (LDV), Days to flowering time under Short Day with vernalization at 4°C during 5 weeks (SDV), Days to FT under LD with vernalization for 0 weeks, 2 weeks, 4 weeks, 8 weeks (0W, 2W, 4W, 8W), Flowering time at 10°C, 22°C (FT10, FT22), leaf number at flowering time at 10°C, 22°C (LN10, LN22) (**Supplementary Data.zip**).

**Figure 1:**
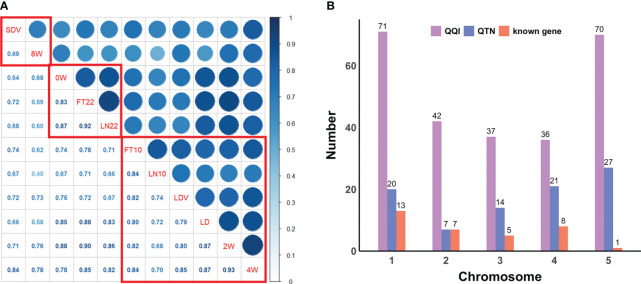
**(A)** Pearson correlation coefficients and correlation clustering of flowering-related traits. The lower diagonal represents the correlation coefficients, and the red boxes indicate the clustering results. **(B)** Distribution of QQIs, QTNs, and known genes across all chromosomes for eleven flowering-related traits.

To explore the relationship among the above flowering-related traits, we computed the Pearson correlation coefficients (PCCs) using the *cor.test* function in R (Version 4.2.1) and generated a phenotypic correlation heatmap using the *ggcorrplot* function from the ggcorrplot package. A hierarchical cluster analysis of the phenotypes was also performed using the *hclust* function in R to divide traits into groups that correlated more significantly into the same group (**Figure 1A**).

### GWAS method

**Figure 2:**
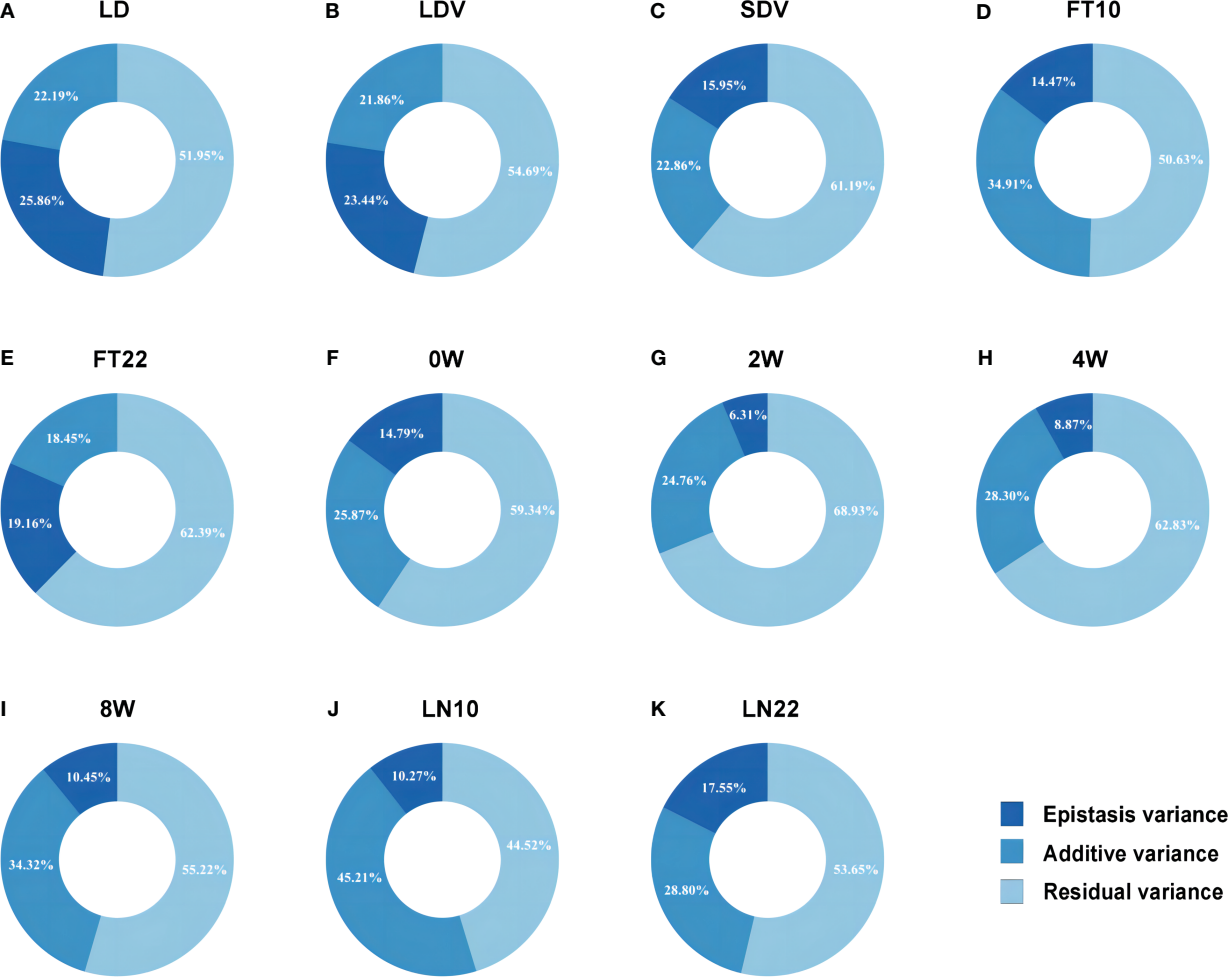
Phenotypic variation explained by the epistatic and additive effects for eleven flowering-related traits. **(A–K)** correspond to the traits LD, LDV, SDV, FT10, FT22, 0W, 2W, 4W, 8W, LN10, and LN22, respectively.

To rapidly and accurately analyze epistasis for GWAS, we combined *Levene’s* test (Brown and Forsythe, 1974) with 3VmrMLM. Firstly, we conducted *Levene’s* test from the OSCA software tool (http://cnsgenomics.com/software/osca; Zhang et al., 2019) for mining the potential epistatic single nucleotide polymorphisms (SNPs) as well as alleviating computational burden. We utilized “*––vqtl -mtd 2*” for *Levene’s* test with median and “*––maf 0.01*” for removing data with minor allele frequency (MAF) < 0.01 in OSCA, resulting in the top 5,000 loci for each trait. Subsequently, we used the *IIIVmrMLM* package (https://github.com/YuanmingZhang65/IIIVmrMLM; Li et al., 2022b) in R to detect QQIs and QTNs, with parameter set to “Epistasis”. 3VmrMLM determines the significance of QQIs or QTNs using either Bonferroni correction (P-value < 0.05/[*m* × (*m*–1)]/2, where *m* is the number of markers) for significant association or a logarithm of odds (LOD) score of 3.0 for suggestive association, either criterion indicates a significant association with the traits. We used *V*_p_ = *V*_epi_ + *V*_add_ + *V*_r_ (**Figure 2**) for each trait to calculate the proportion of the sum of epistatic variance (*V*_epi_) to the phenotypic variance (*V*_p_), where *V*_add_ is the sum of additive variance of detected QTNs and *V*_r_ is the residual variance.

### Identification of known genes

We identified genes located within a 20 kb distance around significant loci, specifically focusing on known genes that have been previously reported in relevant articles. Then the *Arabidopsis* Information Resource (TAIR) (https://www.arabidopsis.org/) and National Center for Biotechnology Information (NCBI) (https://www.ncbi.nlm.nih.gov/) were employed for gene annotation. Known gene mining involved three steps. First, extracting genes within a 20 kb region around significant loci detected by 3VmrMLM from the *Arabidopsis* gene library downloaded from TAIR. Second, screening for genes impacting flowering-related traits and containing relevant keywords. Third, confirming the association between genes and flowering time in *Arabidopsis*, as well as their confirmed epistatic interactions with other genes by retrieving literature from TAIR and NCBI. Finally, known genes will be identified.

### Differential expression and functional enrichment analyses

After excluding known genes reported in the literature, we performed differential expression analysis on the remaining unreported genes using the Gene Expression Omnibus (GEO) database (https://www.ncbi.nlm.nih.gov/geo/). We utilized the GSE197581, GSE190748, and GSE40455 series for targeting differentially expressed genes (DEGs) in response to different temperature, photoperiod, and vernalization treatments. The GSE197581 series included two samples of *Arabidopsis* at 22°C and 10°C, with three biological replicates. The GSE190748 series consisted samples subjected to long-day (16h light/8h dark) and short-day (8h light/16h dark), with two biological replicates. The GSE40455 series included samples to four weeks of vernalization and samples subjected without vernalization treatment, with four biological replicates. For the GSE190748 and GSE40455 series, we used the “*analyze with GEO2R*” tool to identify genes with an absolute log_2_FoldChange greater than 1 and a P-value less than 0.05. For the GSE197581 series, we used the provided data from the website and identify genes with an absolute log_2_FoldChange greater than 1 and the false discovery rate (FDR) less than 0.05. Subsequently, the DEGs obtained above were intersected with the detected unreported genes around QQIs and QTNs, resulting in identification of DEGs associated with flowering-related traits. For gene ontology (GO) based functional enrichment analysis, we submitted the above flowering-related DEGs information to the DAVID platform (https://david.ncifcrf.gov/), and selected the enriched gene ontology terms with a significance threshold of P-value less than 0.05.

### Haplotype analysis for identifying candidate genes

We used the HaploView software (Version 4.1) to perform linkage disequilibrium and haplotype block studies (Barrett et al., 2005) based on the SNPs within these genes and 2 kb upstream of them, which are obtained from GO enrichment analysis. Meanwhile, we employed the *t.test* function in R to examine the phenotypic differences among haplotypes. Candidate genes were identified as those exhibiting significant phenotypic differences across various haplotypes. This approach allowed us to identify potential genes associated with the traits of interest.

## Results

### Phenotypic correlation and clustering

PCCs were obtained from correlation analysis of eleven quantitative traits (**Figure 1A**). The phenotypic correlations of all flowering-related traits showed positive. There were two pairs of PCCs more than 0.90, 2W and 4W (PCCs = 0.93), FT22 and LN22 (PCCs = 0.92), and only one pair of PCCs less than 0.50, LN10 and 8W, but their PCCs also reached 0.48. The above results indicate that eleven traits play an important role in the regulation of flowering time in *Arabidopsis*, and there is a very significant positive correlation between any two pairs.

Hierarchical cluster analysis of all traits by the *hclust* function in R ranked the phenotypes with more significant correlations and divided them into three groups (**Figure 1A**). The first group was SDV and 8W with a correlation coefficient of 0.69; the second group was 0W, FT22, and LN22 with PCCs ranging from 0.83 to 0.92; and the third group was FT10, LN10, LDV, LD, 2W, and 4W with PCCs ranging from 0.68 to 0.93. Clustering of these phenotypes revealed a higher overall correlation between these traits and a greater likelihood of interactions between loci, which was further confirmed following by the pleiotropy of known genes (**Table 1**).

**Table 1:** Pleiotropic genes reported around QQIs/QTNs.

Gene	Bp	Marker	QQI/QTN	Trait	Annotation	Reference
*AGL17* (*AT2G22630*)	chr2:9618207..9622163	SNP66970	QQI	LD	MADs domain containing protein involved in promoting flowering	Han et al., 2008
		SNP66990	QQI	LN22		
		SNP67001	QQI	FT22		
*LUH* (*AT2G32700*)	chr2:13866721..13872246	SNP72705	QTN	2W	WD40 repeat and LUFS domain containing protein that is similar to LUG	Stahle et al., 2009
		SNP72736	QQI	FT22		
		SNP72738	QQI	FT10		
*BOP2* (*AT2G41370*)	chr2:17237727..17240609	SNP77354	QQI	2W	cytoplasmic and nuclear-localized NPR1 like protein	Chahtane et al., 2018
		SNP77376	QQI	LN10		
*ATH1* (*AT4G32980*)	chr4:15914670..15918153	SNP157833 SNP157883	QQI QQI	LDV	increased levels of ATH1 severely delay flowering	Li et al., 2012
				0W		
*CPL3* (*AT4G01060*)	chr4:460395..461246	SNP125917	QTN	2W	Myb-related protein similar to CPC	Zhang and Shen, 2022
		SNP125988	QTN	FT10		

### Epistasis mining using 3VmrMLM

After *Levene’s* test in the raw dataset, 3VmrMLM used in the top 5,000 markers detected 130 QQIs (107 significant and 23 suggested QQIs; **Supplementary Table 1**) and 89 QTNs (61 significant and 28 suggested QTNs; **Supplementary Table 2**) that were strongly associated with the flowering-related traits.

Overall, QQIs and QTNs are distributed on all chromosomes (**Figure 1B**). For QQIs, 3VmrMLM detected a large number of loci, with the highest distribution on chromosome 1 and 5, with 71 and 70 loci, respectively. Although it has a relatively small distribution on chromosomes 2 and 4, it also has more than 35 loci (**Figure 1B**). For QTNs, the distribution of loci on chromosome 2 was relatively uniform, with the number ranging from 14 ~27, except for a minimum of 7 loci on chromosome 2 (**Figure 1B**). On chromosome 1 and chromosome 5, QQIs and QTNs are relatively large, and we can analyze that these two chromosomes have a great influence on the genetic variation of flowering-related traits (**Figure 1B**). In addition, the number of QQIs far exceeded the number of QTNs, indicating that epistasis is a very important link to explore the genetic mechanism of traits related to flowering time, and the interaction between loci is relatively common.

**Figure 3:**
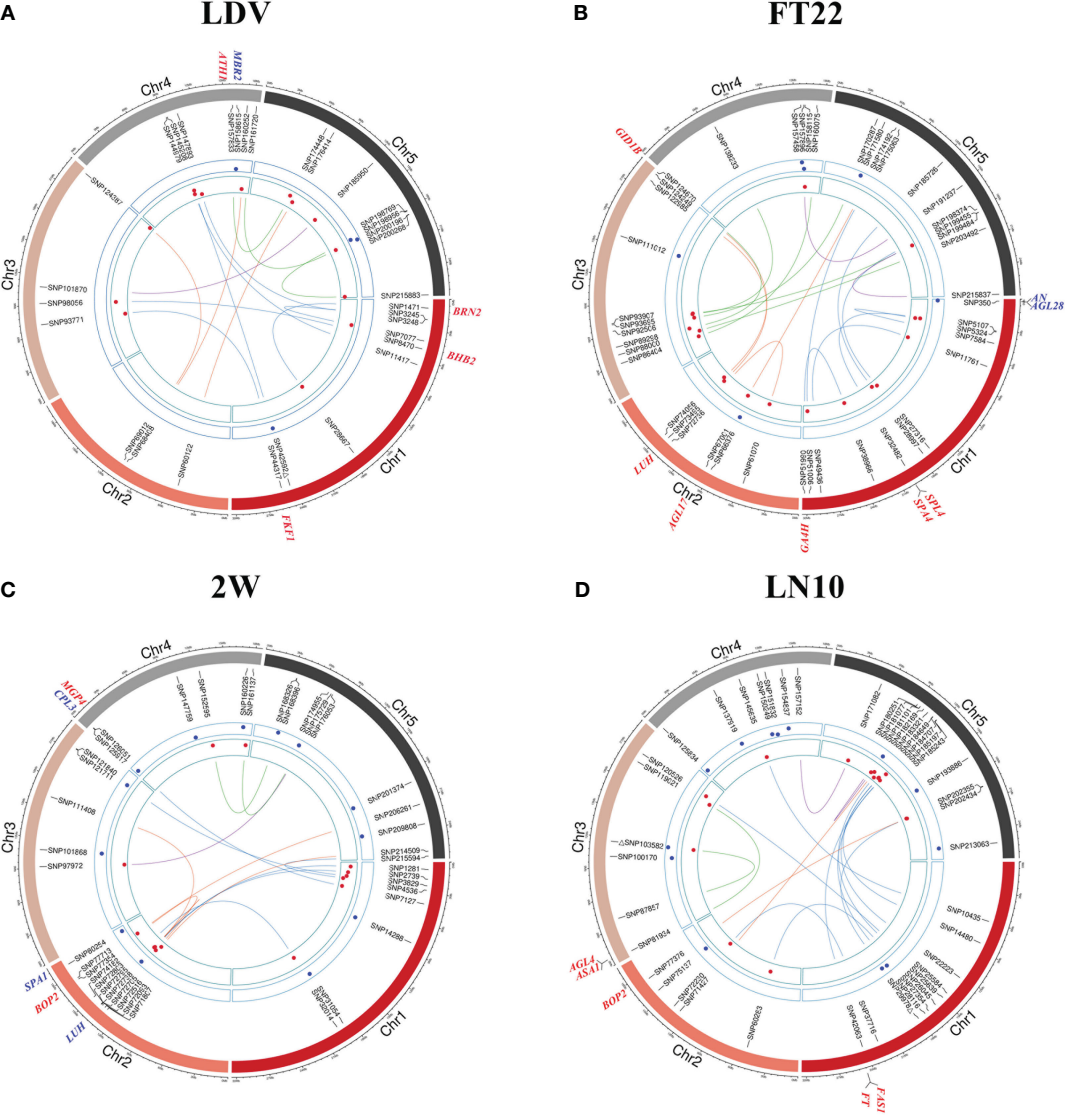
Chord diagrams for QQIs and QTNs detected by 3VmrMLM. **(A–D)** correspond to the traits LDV, FT22, 2W, and LN10, respectively. The inner circle displays the detected QQIs or QTNs (△ indicates overlapping loci between QQIs and QTNs), the height of red dots represents the epistatic effects of QQI pairs, and the height of blue dots represents the additive effects of corresponding QTNs. The outer circle indicates the known genes in vicinity of significant loci.

Six of the 11 traits obtained more than 10 QQIs (**Supplementary Table 1**). FT22 detected the most QQIs, reaching 19 QQIs, with P values of 2.965E-09~1.386E-04, LOD scores of 3.154~7.645, respectively, and 7 positive effects (**Figure 3B**; **Supplementary Table 1**). FT10 detected 11 QQIs with P values of 2.293E-10~ 9.951E-05 and LOD scores of 3.289~8.730, where SNP72738 on chromosome 2 and SNP167863 on chromosome 5 also were the QQIs for 2W and LN22 traits, respectively (**Supplementary Figure 1C**; **Supplementary Table 1**). LN10 detected 16 QQIs, second only to FT22, with P values of 1.327E-10~5.173E-05 and LOD scores of 3.558~8.962, respectively (**Figure 3D**; **Supplementary Table 1**). LN22 detected 10 QQIs, with P values of 6.250E-10~1.190E-04 and LOD scores of 3.216~8.304, respectively (**Supplementary Figure 1G**; **Supplementary Table 1**). LDV detected 14 QQIs, with P values of 4.326E-15~1.174E-04 and LOD scores of 3.221~13.365, 7 positive effects, respectively (**Figure 3A**; **Supplementary Table 1**). SDV detected 14 QQIs, with P values of 4.136E-11~1.379E-04, LOD scores of 3.156~9.457, and 4 positive effects, respectively. Notably, SNP200347 on chromosome 5 was involved in interactions with both SNP179236 and SNP32689. Trait 0W detected 12 QQIs, with P values of 2.605E-14~1.318E-05 and LOD scores of 4.123~12.608, respectively (**Supplementary Figure 1D**; **Supplementary Table 1**). Trait 2W detected 14 QQIs, with P values of 3.985E-09~8.515E-05 and LOD scores of 3.353~7.520, respectively, and SNP72738 was found to be involved in intercrossing with SNP2739 and SNP72795 simultaneously in this trait (**Figure 3C**; **Supplementary Table 1**).

8 QQIs were detected for both 4W and 8W, with P values of 5.906E-13 ~3.681E-06, LOD scores of 4.652~11.266, respectively, and only 2 positive effects for 4W (**Supplementary Figure 1E**; **Supplementary Table 1**). P values of 4.899E-08~1.064E-04 and LOD scores of 3.261~6.462 for 8W (**Supplementary Figure 1F**; **Supplementary Table 1**). Although LD obtained the least number of QQIs, only four, with P values of 2.792E-08~8.968E-07 and LOD scores of 5.242~6.699, respectively, the phenotypic contribution of all four pairs of epistatic loci was >4%, with the pair SNP66960 and SNP71678, located on chromosome 2, having the largest percentage of phenotypic variance explained (PVE) of all QQIs at 8.187%. (**Supplementary Table 1**).

For QTNs, a total of 89 significant/suggestive QTNs were detected to be associated with at least one of the 11 flowering-related traits (**Figure 3**; **Supplementary Figure 1**; **Supplementary Table 2**). Among these QTNs, 3, 4, 8, 10, 6, 6, 13, 11, 11, 13, and 7 QTNs were associated with LD, LDV, SDV, FT10, FT22, 0W, 2W, 4W, 8W, LN10, and LN22, respectively (**Supplementary Table 2**), and the PVE of all QTNs for each trait were 22.193%, 21.875%, 22.864%, 34.906%, 18.446%, 25.868%, 24.760%, 28.297%, 34.328%, 45.205%, and 28.797%, respectively, with P values ranging from 1.757E-10 to 1.986E-04 and LOD scores of 3.006 to 8.843 (**Figure 2**; **Supplementary Table 2**). Notably, SNP31054 and SNP101868 on chromosomes 1 and 3 were involved in both 2W and 4W phenotypic variants, and in addition, SNP103582 on chromosome 3 was detected on both LN10 and FT10 (**Figure 3D**; **Supplementary Figure 1C**; **Supplementary Table 2**).

The total PVE for each trait, considering both additive and epistatic effects, was calculated using the IIIVmrMLM package in R, and the results were visualized in **Figure 2**. The PVE of QQIs for the traits LD, LDV, and FT22 were 25.856%, 23.438%, and 19.163%, respectively, as shown in **Figures 2A, B, E**. Accordingly, these values were higher than the PVEs of the corresponding QTNs. The analysis of QQIs and QTNs revealed that most locus exhibited either epistatic or additive effects in contributing to phenotypic variation of each trait (**Figure 2**; **Supplementary Tables 1**, **2**). However, we also identified some specific SNPs, such as SNP42592 for LDV, both SNP103582 and SNP29978 for LN10, SNP200347 for SDV, SNP125854 for 0W, SNP101868 for 4W, both SNP111498 and SNP181717 for 8W, which were involved in both additive and epistatic effects (**Figures 3A, D**; **Supplementary Figures 1B, D, E, F**; **Supplementary Tables 1**, **2**).

### Known genes around QQIs and QTNs for flowering-related traits in *Arabidopsis*

TAIR (https://www.arabidopsis.org/) was used to mine the known genes around QQIs and QTNs (20 kb upstream and downstream of each locus). A total of 34 known genes were found to be located around the significant/suggested loci, including 29 QQIs and 12 QTNs (**Figure 3**; **Supplementary Figure 1**; **Supplementary Table 3**).

For QQIs, 3, 4, 2, 1, 6, 4, 2, 0, 1, 5, and 1 known genes were explored in LD, LDV, SDV, FT10, FT22, 0W, 2W, 4W, 8W, LN10, and LN22, respectively (**Supplementary Table 3**). Specifically, the known genes *BRN2* (*AT1G03457*, near SNP1471) and *FKF1* (*AT1G68050*, near SNP44317) associated with LDV (**Figure 3A**; **Supplementary Table 3**) interact with the *AtBRN* and *CDF2* protein to promote or repress flowering in *Arabidopsis*, respectively (Kim et al., 2013; Lee et al., 2018). The known gene *SPA4* (*AT1G53090*) associated with FT22 is located near SNP32482 (**Figure 3B**; **Supplementary Table 3**). There has been reported that *SPA4* is involved in regulating photoperiodic flowering in *Arabidopsis* and interacts with the flower inducer CO to regulate flowering stability, while it interacts with *PAP1* and *PAP2* and is involved in repressive regulation at the transcriptional level, affecting light conditions growth of *Arabidopsis* under light conditions (Laubinger et al., 2006; Maier et al., 2013). Two known genes, *FT* (*AT1G65480*) and *FAS1* (*AT1G65470*), were detected simultaneously near SNP42063 (**Figure 3D**; **Supplementary Table 3**), and two known genes, *ASA1* (*AT3G02260*) and *AGL4* (*AT3G02310*), were detected near SNP81934 under LN10 (**Figure 3B**; **Supplementary Table 3**), where *FT* interacts with *FD(AT4G35900)* and 14-3-3 proteins to produce a florigen-activation complex, control flowering time, and correct the expression of floral homologs to promote flowering (Collani et al., 2019); the known gene *AGL4* interacts with DNA and may be involved in forming a tetrameric DNA-binding complex to control flower development and thus affect flowering time (Jetha et al., 2014). The known gene *HOS1* (*AT2G39810*, near SNP76337) associated with trait 0W (**Supplementary Figure 1D**; **Supplementary Table 3**) is localized to the nuclear membrane and interacts with *Nup96*, and loss of function of *Nup96* would lead to disruption of *HOS1* protein, resulting in excessive accumulation of CO protein, a key activator of flowering under long-day that suppresses early flowering in *Arabidopsis* under long-day (Lazaro et al., 2015).

For QTNs, 1, 2, 1, 2, 3, 1, and 2 known genes were explored in LDV, SDV, FT10, FT22, 2W, 4W, and LN22, respectively, and only QQI-related genes were obtained for the remaining four traits (**Supplementary Table 3**). Among the significant loci associated with SDV, *FD* (*AT4G35900*) was found to be located near SNP159681 (**Supplementary Figure 1B**; **Supplementary Table 3**), and it was shown that *FD* acts as a transcriptional activator of floral tissue identity genes to regulate flowering time in *Arabidopsis*, while the *FD* transcription factor was shown to interact with *TFL1* by BiFC assay to induce flowering time and inflorescence meristem tissue by *Arabidopsis* repressor genes development is fine-tuned (Hanano and Goto, 2011; Gorham et al., 2018). In the case of FT22, two known genes, *AN* (*AT1G01510*) and *AGL28* (*AT1G01530*), were detected simultaneously near SNP350 (**Figure 3B**; **Supplementary Table 3**), and *AN* has been shown to control leaf morphology and thus indirectly affect flowering time in *Arabidopsis*. (Stern et al., 2007); *AGL28* can act as a flower activator by up-regulating the expression of known flower promoters within the autonomous pathway, and its overexpression will up-regulate the expression of *FCA* and *LUMINIDEPENDENS*, leading to early flowering in *Arabidopsis* (Yoo et al., 2006). One known gene associated with LDV, *MBR2* (*AT4G34040*), located near SNP158615 (**Figure 3A**; **Supplementary Table 3**), was shown in earlier studies to promote flowering through a *PFT1* dependent and independent mechanism (Iñigo et al., 2012). The gene *SPA1* (*AT2G46340*, near SNP80254) is known to be associated with 2W (**Figure 3C**; **Supplementary Table 3**), and is a key repressor of light signaling in the ovary to regulate flowering time by regulating the photoperiod (Ranjan et al., 2011). Near the QTN SNP135761, which is significantly associated with LN22, *CRY1* (*AT4G08920*; **Supplementary Figure 1G**; **Supplementary Table 3**) is known to mediate blue light to promote flowering in *Arabidopsis*, which is more sensitive to flowering photoperiod under blue light, suggesting that *CRY1* plays an important role in flowering regulation (Mockler et al., 2003).

Interestingly, out of these 34 known genes, five pleiotropic genes were involved in the performance variation of at least two traits in terms of QQI or QTN (**Table 1**). In terms of QQI, the known gene *AGL17* (*AT2G22630*), which was detected around SNP67001, SNP66970, and SNP66990 and was associated with FT22, LD, and LN22 (**Table 1**; **Figure 3B**; **Supplementary Figures 1A, G**), has been confirmed to play a role in the photoperiodic pathway of *Arabidopsis* and is positively controlled by the photoperiodic pathway regulator CO. It can promote the flowering of *Arabidopsis thaliana* (Han et al., 2008). At the same time, the known gene *ATH1* (*AT4G32980*, around SNP157833), which is related to LDV and 0W (**Table 1**; **Figure 3A**; **Supplementary Figure 1D**), is necessary for controlling the morphology of *Arabidopsis* flower stalk. In addition, there is an interaction between *ATH1* and *KNAT2*, and the protein complex plays a role in regulating flower pedicle development (Li et al., 2012). *BOP2* (*AT2G41370*), detected near SNP77354 and SNP77376, is associated with two traits, 2W and LN10 (**Table 1**; **Figures 3C, D**), and studies have shown that the *LFY* and *BOP2* proteins physically interact to inhibit bracteal formation and reduce flowering time in a short period of time under certain conditions (Chahtane et al., 2018). In terms of QTN, a known gene *CPL3*(*AT4G01060*, near SNP125917 and SNP125988) was detected to have additive effects on both 2W and FT10 (**Table 1**; **Figure 3C**; **Supplementary Figure 1C**), and *CPL3* gene has pleiotropic effects on flowering development and epidermal cell size of *Arabidopsis* by regulating internal duplication (Zhang and Shen, 2022).

Notable is, known gene *LUH* (*AT2G32700*), located near SNP72736, SNP72705, and SNP72738, exhibited associations with FT22, 2W, and FT10 (**Table 1**; **Figures 3B, C**; **Supplementary Figure 1C**). Furthermore, it displayed both additive and epistatic effects (**Table 1**; **Figures 3B, C**; **Supplementary Figure 1C**). *LUH* showed epistatic effect at FT10 and FT22, and additive effect at 2W. It was shown that *LUH* interacts with *YAB* to regulate distal axis pattern, lateral organ growth, and inflorescence foliation. At the same time, its leaf-based signaling pathway promotes paraxial cell identity in leaves and initiation and maintenance of embryo bud apical meristem SAM (Stahle et al., 2009). More detailed information about the genes surrounding QTNs and QQIs identified by 3VmrMLM can be found in **Supplementary Table 3**.

### Response to different treatments and GO enrichment pathway

**Figure 4:**
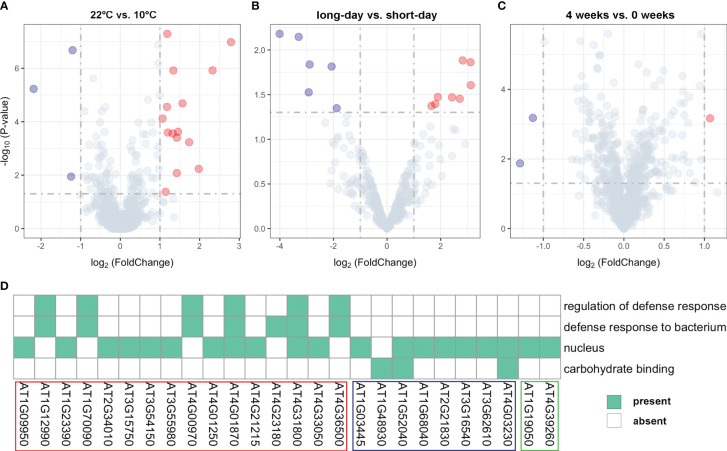
Volcano plots for expression values of **(A)** 18 genes under different temperature treatments (22°C vs. 10°C), **(B)** 14 genes under different photoperiod treatments (long-day vs. short-day), and **(C)** 3 genes under different vernalization time treatments (4 weeks vs. 0 weeks). **(D)** Results of functional enrichment analysis based on gene ontology. The genes highlighted within the red, blue, and green boxes belong to the group of significant DEGs between 22°C vs. 10°C treatments, long-day vs. short-day treatments, and 4 weeks vs. 0 weeks treatments, respectively.

We conducted a comprehensive analysis of gene expression changes under different treatments to gain insights into their responses. Through differential expression analysis on the unreported genes, we successfully identified distinct expression patterns of the 35 genes (**Supplementary Table 4**). Specifically, we found 18 genes that exhibited significant differential expression between 22°C and 10°C treatments (**Figure 4A**; **Supplementary Table 4**), 15 were significantly upregulated at 10°C, while only three genes showed significant downregulation at this temperature. For instance, *AT3G55980*, located near the SNP120225 locus associated with LN22, exhibited a log_2_FoldChange of 2.79 and a P-value of 1.05E-07, as illustrated in the upper right corner of the volcano plot. This gene was found to be enriched in the nucleus (**Figure 4A**; **Supplementary Table 4**). Similarly, 14 genes showed significant differential expression between long-day and short-day treatments (**Figure 4B**; **Supplementary Table 4**), suggesting their involvement in light-dependent processes. Specifically, eight genes exhibited significant upregulation under short-day treatments, while six genes were significantly upregulated under long-day treatments. Additionally, we observed differential expression in 3 genes between 4 weeks and 0 weeks treatments (**Figure 4C**; **Supplementary Table 4**), highlighting their role in a time-dependent response. These findings offer valuable insights into the biological underpinnings of the newly identified genes associated with flowering-related traits in *Arabidopsis*.

To gain further functional insights, we performed GO functional enrichment analysis on the identified DEGs. This analysis revealed that out of the 35 DEGs, 26 genes were significantly enriched in 4 distinct GO terms associated with various biological processes (**Figure 4D**). Furthermore, it was shown that 20 genes located in proximity to QQIs and QTNs were specifically enriched in the nucleus (GO:0005634) (**Figure 4D**). For example, *AT3G55980*, known as *AtSZF1*, has been reported to be associated with the nucleus and is involved in the *Arabidopsis* salt stress response (Sun et al., 2007). Notably, *AT4G01870* and *AT4G31800* were found to be simultaneously associated with three important biological processes (**Figure 4D**). Specifically, *AT4G31800*, known as *WRKY18*, enhances developmentally regulated defense responses in transgenic plants without causing significant negative effects on plant growth (Pandey et al., 2010). On the other hand, *AT4G01870* is involved in the chemical reactions and pathways leading to the synthesis of camalexin, an indole phytoalexin (https://www.arabidopsis.org/). In addition, we observed three genes *AT1G52040*, *AT4G03230*, and *AT1G48930* related to carbohydrate binding (**Figure 4D**), with *AT1G48930* possessing a carbohydrate-binding structural domain (CBM49) that plays a role in *Arabidopsis* root hair and endosperm development, among other functions (del Campillo et al., 2012). Interestingly, we identified a pair of QQIs, *AT1G09950* and *AT1G12990*, in close proximity to the SNP5324 and SNP7584 loci, respectively (**Table 2**). *AT1G09950* is involved in cellular components. It affects seed germination and early seedling growth by increasing sensitivity to abscisic acid (Ren et al., 2010). Meanwhile, *AT1G12990* is associated with the regulation of the defense response (GO:0031347) and the defense response against bacteria (GO:0042742) for glycosyltransferase activity (https://www.arabidopsis.org/).

**Table 2:** Results of 20 candidate genes and functional annotation.

Trait	QQI/QTN	Marker	Candidate Gene	Bp	Annotation	
LDV	QQI	SNP1471	*AT1G03445*	chr1:854410..859701	erine–threonine protein phosphatase	
	QQI	SNP11417	*AT1G19050*	chr1:6577833..6579314	two-component response regulator	
	QQI	SNP44317	*AT1G68040*	chr1:25502864..25505263	S-adenosyl-L-methionine-dependent methyltransferases superfamily protein.	
	QQI	SNP124387	*AT3G62610*	chr3:23154630..23156585	regulates flavonol biosynthesis.	
	QQI	SNP161720	*AT4G39260*	chr4:18273829..18275216	verprolin	
SDV	QQI	SNP66659	*AT2G21830*	chr2:9303713..9306025	encodes a putative DegP protease.	
	QQI	SNP128333	*AT4G03230*	chr4:1418841..1423337	G-type lectin S-receptor-like Serine/Threonine-kinase.	
	QTN	SNP90818	*AT3G16540*	chr3:5626290..5628857	encodes a putative DegP protease.	
FT10	QQI	SNP126845	*AT4G01870*	chr4:808376..810611	tolB protein-like protein	
	QTN	SNP126164	*AT4G01250*	chr4:522530..524249	involved in regulation of dark induced leaf senescence.	
FT22	QQI	SNP5324	*AT1G09950*	chr1:3240531..3241863	response to aba and salt 1	
	QQI	SNP7584	*AT1G12990*	chr1:4433605..4436102	beta-1,4-N-acetylglucosaminyltransferase family protein	
	QQI	SNP73495	*AT2G34010*	chr2:14368536..14370438	verprolin	
LN10	QQI	SNP14480	*AT1G23390*	chr1:8308965..8310916	kelch domain-containing F-box protein	
	QQI	SNP119021	*AT3G54150*	chr3:20050564..20052931	S-adenosyl-L-methionine-dependent methyltransferases superfamily protein	
	QTN	SNP125834	*AT4G00970*	chr4:418327..421885	encodes a cysteine-rich receptor-like protein kinase.	
	QTN	SNP151832	*AT4G23180*	chr4:12137995..12140930	encodes a receptor-like protein kinase.	
LN22	QQI	SNP45945	*AT1G70090*	chr1:26400694..26402815	encodes a protein with putative galacturonosyltransferase activity.	
	QQI	SNP90174	*AT3G15750*	chr3:5334844..5336485	essential protein Yae1	
	QQI	SNP120225	*AT3G55980*	chr3:20776220..20778952	CCCH-type zinc finger protein involved in salt stress and immune responses.	

### Haplotype and phenotypic difference analysis of candidate genes

To further validate the association between genes and flowering-related traits, we performed haplotype analysis on the SNPs within the 2 kb upstream regions of the 26 genes identified from the GO enrichment analysis. In total, 20 candidate genes were identified, which significant phenotypic differences were observed among their haplotypes (**Table 2**). These genes were associated with six different traits, namely LDV, SDV, FT10, FT22, LN10, and LN22 (**Table 2**). Among them, 16 genes were located near QQIs, while 4 genes were located near QTNs. It is worth noting that the loci near *AT1G03445* and *AT1G68040*, which correspond to these genes, also contain previously reported known genes. More detailed information was listed in **Table 2**; **Supplementary Table 5**.

**Figure 5:**
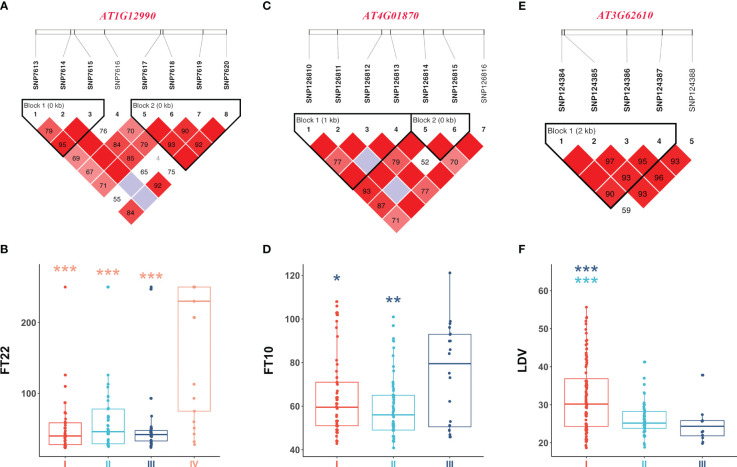
Linkage disequilibrium and haplotype block analysis for the candidate genes **(A)** *AT1G12990* associated with FT22, **(C)** *AT4G01870* associated with FT10, and **(E)** *AT3G62610* associated with LDV, respectively. **(B)** Comparison of FT22 across various haplotypes I (CGGGGTG), II (CGGGTTG), III (TGGGTTG), and IV (TGTGTTT). **(D)** Comparison of FT10 across various haplotypes I (GTCTGG), II (TTGTTG), and III (TTGTTT). **(F)** Comparison of LDV across various haplotypes I (AAAG), II (AGTA), and III (CGTA). In the boxplots, the center line represents the median, the box limits indicate the upper and lower quartiles, and the whiskers extend 1.5 times the interquartile range. Data points beyond the whiskers are considered outliers and plotted individually. The number of stars indicates the significance level from *t-*test (*0.05, **0.01, ***0.001).

**Figure 5** illustrates the analysis of *AT1G12990* (CDS coordinates [5’-3’]: 4433605-4436102), *AT4G01870* (CDS coordinates [5’-3’]: 808376-810611), and *AT3G62610* (CDS coordinates [5’-3’]: 23154630-23156585) to reveal intragenic variations impacting flowering time and identify favorable haplotypes. **Figure 5A** presents the linkage disequilibrium and haplotype block with 8 SNPs for the gene *AT1G12990*, located near the SNP7584 locus, a QQI for FT22 (**Table 2**). After removing 53 missing values from the phenotypic data, the remaining 146 individuals were classified into four haplotypes based on seven SNPs (SNP7613, SNP7614, SNP7615, SNP7617, SNP7618, SNP7619, and SNP7620). Haplotype IV (TGTGTTT) exhibited significantly higher median phenotypic values for FT22 compared to the other three haplotypes (**Figure 5B**). Haplotype IV consisted 25 individuals, among which 12 had a maximum phenotypic value of 250 for the FT22 trait, while the other three haplotypes had values of 1, 4, and 1, respectively. Additionally, a *t*-test demonstrated significant differences between haplotype IV and haplotypes I (CGGGGTG, P-value = 5.65E-07), II (CGGGTTG, P-value = 9.16E-06), and III (TGGGTTG, P-value = 7.98E-07; **Supplementary Table 5**). Similarly, the candidate gene *AT1G09950* (CDS coordinates [5’-3’]: 4433605-4436102), located near the SNP5324 locus, showed an interaction effect with the SNP7584 locus for the FT22 trait. **Supplementary Figure 2A** depicts the linkage disequilibrium and haplotype block analysis using 11 SNPs. After removing 42 missing values from the phenotype data, the remaining 157 individuals were divided into three haplotypes based on seven SNPs (SNP5265, SNP5266, SNP5267, SNP5268, SNP5269, SNP5271, and SNP5272). **Supplementary Figure 2B** demonstrates significant differences between haplotype I (ATATAGT) and haplotype III (GAGGTCT, P-value = 1.73E-02; **Supplementary Table 5**). Therefore, we inferred that the candidate genes *AT1G12990* and *AT1G09950* may interact with each other and play a role in flowering time regulation in *Arabidopsis*.

**Figures 5C, D** present the haplotype block and phenotype differences of the candidate gene *AT4G01870*, detected around the SNP126845 locus, a QQI for FT10 (**Table 2**; **Supplementary Table 5**). Haplotype III (TTGTTT) exhibited the highest median phenotypic values and showed significant differences with haplotype I (GTCTGG, P-value = 4.20E-02) and haplotype II (TTGTTG, P-value = 6.87E-03; **Supplementary Table 5**). Similarly, the candidate gene *AT3G62610* was detected around the SNP124387 locus, a QQI for LDV (**Table 2**; **Supplementary Table 5**). **Figures 5E, F** illustrate the haplotype block and phenotype differences. Hence, we suggest that the candidate genes *AT4G01870* and *AT3G62610* may influence the flowering time in *Arabidopsis*.

Additionally, the candidate gene *AT4G01250* (CDS coordinates [5’-3’]: 522530-524249) was detected around the SNP126164 locus, a QTN for FT10, while the candidate gene *AT4G00970* (CDS coordinates [5’-3’]: 418327-421885) was detected near the SNP125834 locus, a QTN for LN10 (**Table 2**; **Supplementary Table 5**). **Supplementary Figures 2C–F** display the haplotype block and phenotype differences of these two genes. We hypothesize that the candidate genes *AT4G01250* and *AT4G00970* may also affect the flowering time in *Arabidopsis*.

In summary, we propose that the four candidate genes mentioned above, located near QQIs, may exert potential influence on their corresponding traits, among them *AT1G12990* and *AT1G09950* might have gene-by-gene interaction. Furthermore, several candidate genes near QTNs exhibited significant differences in phenotypes across haplotypes (**Supplementary Table 5**). However, further experimental verification is required to determine whether these candidate genes interact with each other in regulating flowering in *Arabidopsis*.

## Discussion

### *Levene’s* test for potential epistasis

Due to the substantial computational requirements in QQI detection, particularly when considering the population structure and polygenic backgrounds in 3VmrMLM, it is advisable to limit the number of markers to less than 5,000 (Li et al., 2022a; Li et al., 2022b). To obtain the potential epistasis and alleviate the computational burden, we employed *Levene’s* test, which can be used to detect potential loci for heterogeneity of variances due to potentially interacting SNPs such as QTN-by-QTN interactions (Zhang et al., 2019). However, the direct application of *Levene’s* test to the raw data did not reveal any significant interacting loci due to the large number of markers and the stringent threshold of the Bonferroni correction. Moreover, potential limitations of *Levene*’s test include no covariates are allowed and only equality of variances, but not means, can be tested (Dumitrascu et al., 2019), that is, it could neither consider the population structure nor obtain the effect estimate of markers. Therefore, for each trait, we firstly selected the top 5,000 significantly associated variance-controlling SNPs detected by *Levene’s* test, which also exhibited that P values were less than 0.05, and then performed QQI detection of 3VmrMLM using these top 5,000 loci for input. Combining potential epistasis loci selection with 3VmrMLM significantly improves detection accuracy while greatly reducing computation time.

### Genetic basis for flowering-related traits in *Arabidopsis*

3VmrMLM detected 130 QQIs and 89 QTNs significantly associated with 11 flowering-related traits in the analysis of epistasis. Among them, the PVE of QQIs for the traits LD, LDV, and FT22 were 25.856%, 23.438%, and 19.163%, respectively (**Figures 2A, B, E**), which were higher than those of QTNs at 22.193%, 21.863%, and 18.446% (**Figures 2A, B, E**), indicating that QQIs contribute more to phenotypic variation than QTNs for these three traits and epistasis is a non-negligible factor contributing to phenotypic variation. Notably, A pair of loci SNP66960 and SNP71678, located on chromosome 2 under LD, had the highest PVE among all traits in terms of QQI, at 8.187% (**Supplementary Table 1**). In its vicinity, the known gene *SVP* (*AT2G22540*; **Supplementary Figure 1A**; **Supplementary Table 3**) has been shown to be an important regulator during the transition to flowering and floral development, while *SVP* interacts with *OsMADS22* and *OsMADS47* to interfere with normal *Arabidopsis* flower development (Fornara et al., 2008).

The known genes *BRN2* (*AT1G03457*) located near QQI SNP1471 (P-value = 4.32628E-15, LOD = 3.2212) and *FKF1* (*AT1G68050)* located near QQI SNP44317 (P-value = 1.37721E-07, LOD = 5.8963; **Figure 3A**; **Supplementary Table 3**) are both associated with LDV and interact with *AtBRN*, CDF2 protein to promote or repress flowering in *Arabidopsis*, respectively (Kim et al., 2013). The known gene *SPA4* (*AT1G53090*) associated with FT22 is located near QQI SNP32482 (P-value=1.35181E-08, LOD=7.0044; **Figure 3B**; **Supplementary Table 3**). *SPA4* is involved in regulating *Arabidopsis* photoperiodic flowering and was found to interact with both CO, *PAP1* and *PAP2* to jointly regulate flowering stability and growth under light conditions (Laubinger et al., 2006; Maier et al., 2013).Two known genes, *FT* (*AT1G65480*) and *FAS1* (*AT1G65470*), were detected simultaneously near QQI SNP42063 (P-value=9.97104E-07, LOD=5.6226) under the LN10 trait (**Figure 3D**; **Supplementary Table 3**), where *FT* interacts with *FD* (*AT4G35900*), and 14-3-3 proteins interact to produce florigen-activation complex to control flowering time and correct expression of floral homologs and promote flowering (Collani et al., 2019). On the other hand, the known genes with QTN effects *FD* (*AT4G35900*, near QTN SNP159681; Hanano and Goto, 2011; Gorham et al., 2018), *AGL28* (*AT1G01530*, near QTN SNP350; Yoo et al., 2006), *MBR2* (*AT4G34040*, near QTN SNP158615; Iñigo et al., 2012) and 8 other genes have been reported to influence flowering through different pathways to exert either facilitative or repressive effects on flowering (**Figure 3**; **Supplementary Figure 1**; **Supplementary Table 3**).

Note that we also uncovered five pleiotropic known genes that act on multiple traits in terms of QQI or QTN. The known gene *AGL17* (*AT2G22630*), detected around QQI SNP67001, SNP66970, and SNP66990, is associated with three traits FT22, LD, and LN22 (**Table 1**; **Figure 3B**; **Supplementary Figures 1A, G**). It has been shown to be positively regulated by the photoperiod pathway regulator CO to promote flowering in *Arabidopsis* (Han et al., 2008). The known genes *ATH1* (*AT4G32980*, around QQI SNP15783; **Table 1**; **Figure 3A**; **Supplementary Figure 1D**) associated with LDV and 0W are required for the control of *Arabidopsis* flower stem morphology and interact with *KNAT2* to help regulate flower tip development (Li et al., 2012). *BOP2* (*AT2G41370*) was detected around QQI SNP77354 and QQI SNP77376 were detected in the vicinity, associated with 2W and LN10 (**Table 1**; **Figures 3C, D**), and BOP2 proteins interaction with *LFY* has been reported to shorten flowering time in a short period of time (Chahtane et al., 2018). The known gene *CPL3* (*AT4G01060*, around QTN SNP125988 and QTN SNP125917) was detected to have additive effects on both FT10 and 2W (**Table 1**; **Figure 3C**; **Supplementary Figure 1C**), confirming a pleiotropic effect on flowering development in *Arabidopsis* (Zhang and Shen, 2022). The known gene *LUH* (*AT2G32700*, around QQI SNP72736, QTN SNP72705, and QQI SNP72738) was uncovered to be involved not only in three traits FT22, 2W, and FT10, but also found to have additive and epistatic effects (**Table 1**; **Figures 3B, C**; **Supplementary Figure 1C**), and studies showed that *LUH* interacts with *YAB* and plays a regulatory role on lateral organ growth and inflorescence leaf management (Stahle et al., 2009). The phenotypic association results of *BOP2* (*AT2G41370*) and *CPL3* (*AT4G01060*) were consistent with the phenotypic clustering results shown in **Figure 1A**. Additionally, the traits LN22 and FT22 associated with *AGL17* (*AT2G22630*), as well as the traits 2W and FT10 associated with *LUH* (*AT2G32700*), were also grouped together (**Figure 1A**; **Table 1**). These findings further support the reliability of our analysis.

Except for known genes, we also identified 20 candidate genes in this study (**Table 2**). Among them, *AT1G12990*, *AT1G09950*, *AT4G01870*, and *AT3G62610*, located near QQIs, specially, former two genes showed potential gene-by-gene interactions related to flowering traits in *Arabidopsis*. Specifically, *AT1G12990* was found in proximity to the SNP7584 locus, while *AT1G09950* was found near the SNP5324 locus, and remarkably, these loci coincided with a significant pair of QQIs associated with the trait FT22 (P-value = 7.08064E-05, LOD = 3.4287; **Supplementary Table 1**). *AT4G01870* was detected near the SNP126845 locus, forming a QQI with SNP185421 for FT10 (P-value = 5.12209E-08, LOD = 6.443; **Supplementary Table 1**). Additionally, *AT3G62610* was found around the SNP124387 locus, forming a QQI with SNP69012 for LDV (P-value = 4.70143E-06, LOD = 4.5505; **Supplementary Table 1**). These candidate genes also showed differential expression under 22°C vs. 10°C and long-days vs. short-days treatments (**Figures 4B, C**; **Supplementary Table 4**). *AT1G12990* and *AT4G01870* were associated with the regulation of defense response (GO:0031347) and defense response to bacterium (GO:0042742), while *AT1G09950*, *AT4G01870*, and *AT3G62610* were involved in nucleus-related functions (GO:0005634). Notably, significant phenotypic differences were observed across different haplotypes. Therefore, we hypothesize that these candidate genes, namely *AT1G12990*, *AT1G09950*, *AT4G01870*, and *AT3G62610*, in proximity of QQIs, may play a role in influencing flowering in *Arabidopsis*. Specially, *AT1G12990* and *AT1G09950* might exist potential gene-by-gene interaction. However, further experimental validation, such as functional validation, is necessary to explore these gene-by-gene interactions for flowering-related traits.

### Methods comparison

To better analyze the QQIs results obtained from the 3VmrMLM method, we performed epistasis analysis in the raw dataset using PLINK (Purcell et al., 2007). The command used for detecting pairs of epistatic loci was “*plink --file genotype --pheno phenoq.txt --epistasis --epi1 P-value --allow-no-sex --out result*”, with a threshold using Bonferroni correction. The number of significant interacting loci detected for each trait using PLINK ranged from 2,903 to 41,132 (**Supplementary Table 6**). It is well-known that PLINK uses a simple linear model, which computes quickly even with large sample sizes, but it does not consider the polygenic background, leading to an increased false positive rate (Purcell et al., 2007). In addition, except for trait 0W, the number of significant QQIs detected by PLINK that overlap with those detected by 3VmrMLM ranged from 1 to 34. Among them, for trait FT22, PLINK detected a total of 41,132 QQIs, out of which 34 were simultaneously detected by 3VmrMLM (**Supplementary Table 6**). This suggests that QQIs detected by 3VmrMLM are likely to be potential interacting loci.

We also employed the REMMA method (Ning et al., 2018), a mixed linear model-based approach, for conducting epistasis analysis in the raw dataset. This method incorporates both additive and dominance relationship matrices, offering theoretical control over Type I errors when examining pairwise epistatic SNPs. Among the eleven traits, three (SDV, FT22, and 8W) showed significant interacting loci, with 429, 72, and 3,541 loci detected, respectively (**Supplementary Table 6**). The QQIs associated with SDV overlapped with those detected by 3VmrMLM (**Supplementary Table 6**).

Similarly, we employed the QMDR approach (Yu et al., 2015) based on machine learning to analyze epistasis. Because no results were obtained in the raw dataset due to the large number of markers and strict Bonferroni correction threshold. Thus, the strategy for top 5,000 marker selection and LOD scores greater than 3.0 was identical to that described for 3VmrMLM in order to be comparable. As listed in **Supplementary Table 6**, only six traits (LD, SDV, FT22, LN22, 4W, and 8W) showed significant interaction loci, while the remaining traits did not. Overall, 3VmrMLM excels in both efficiency and accuracy when analyzing epistasis.

## Conclusion

In this study, we performed the novel 3VmrMLM method in GWAS to investigate the epistatic association with eleven flowering-related traits in *Arabidopsis*. A total of 130 pairs of QQIs and 89 QTNs were successfully detected. Furthermore, through genome annotation and previous research, 29 known genes around QQIs and 12 known genes around QTNs were identified. Among the above known genes, five genes, namely *AGL17* (*AT2G22630*), *ATH1* (*AT4G32980*), *BOP2* (*AT2G41370*), *CPL3* (*AT4G01060*), and *LUH* (*AT2G32700*), were demonstrated an epistatic or additive effect for at least two traits. Moreover, 16 candidate genes around QQIs and 4 candidate genes around QTNs were validated using differential expression analysis, functional enrichment analysis, and haplotype and phenotypic difference analysis. Notably, *AT1G12990* and *AT1G09950* around QQIs exhibited potential gene-by-gene interactions influencing flowering. These findings contribute to the identification and exploration of epistasis associated with flowering-related traits in *Arabidopsis*.

## Data availability statement

The datasets presented in this study can be found in online repositories. The names of the repository/repositories and accession number(s) can be found in the article/**Supplementary Material**.

## Author contributions

LH: Data curation, Formal analysis, Investigation, Validation, Visualization, Writing – original draft. BS: Data curation, Formal analysis, Investigation, Validation, Visualization, Writing – original draft. XW: Data curation, Resources, Writing – review & editing. JZ: Writing – review & editing, Funding acquisition. Y-JW: Conceptualization, Funding acquisition, Supervision, Writing – review & editing.

## Supplementary material

Supplementary Figure 1 Chord diagrams for QQIs and QTNs detected by 3VmrMLM. **(A~G)** correspond to the traits LD, SDV, FT10, 0W, 4W, 8W, and LN22, respectively. The inner circle displays the detected QQIs or QTNs (△ indicates overlapping loci between QQIs and QTNs), the height of red dots represents the epistatic effects of QQI pairs, and the height of blue dots represents the additive effects of corresponding QTNs. The outer circle indicates the known genes in vicinity of significant loci.

Supplementary Figure 2 Linkage disequilibrium and haplotype block for the candidate gene **(A)** *AT1G09950* associated with FT22, **(C)** *AT4G01250* associated with FT10, and **(E)** *AT4G00970* associated with LN10. **(B)** Comparison of FT22 across various haplotypes I (ATATAGT), II (GAGGACT), and III (GAGGTCT). **(D)** Comparison of FT10 across various haplotypes I (TATACTATCT), II (TGGACCATCA), III (TGGACTAAAT), and IV (TGGACTATCT). **(F)** Comparison of LN10 across various haplotypes I (AGCCCACTGA), II (AGCTCGCCGT), III (CAATCGCCGT), and IV (CAATGGCCCT). For boxplots, center line shows median, box limits indicate upper and lower quartiles, and whiskers extend 1.5 times the interquartile range, while data beyond the end of the whiskers are outlying points that are plotted individually. The number of stars represents the result of *t* test at different significance levels (*: 0.05, **: 0.01, ***: 0.001).

Supplementary Data Sheet All the phenotypic values of the traits and all the marker genotypes, which are derived from Atwell et al. Nature 2010; 465(7298), 627-631.

## Glossary

**Table:**

0W	Days to FT under LD with vernalization for 0 weeks
2W	Days to FT under LD with vernalization for 2 weeks
4W	Days to FT under LD with vernalization for 4 weeks
8W	Days to FT under LD with vernalization for 8 weeks
AI	artificial intelligence
BLUP	best linear unbiased prediction
DEGs	differentially expressed genes
FT10	Flowering time at 10°C
FT22	Flowering time at 22°C
FDR	false discovery rate
GWAS	genome-wide association studies
GEO	Gene Expression Omnibus
GO	gene ontology
LD	Days to flowering time under Long Day
LDV	Days to flowering time under Long Day with vernalization at 4°C during 5 weeks
LOD	logarithm of odds
LN10	leaf number at flowering time at 10°C
LN22	leaf number at flowering time at 22°C
MAF	minor allele frequency
MDR	multifactor dimensionality reduction
MLM	mixed linear model
NCBI	National Center for Biotechnology Information
PCCs	Pearson correlation coefficients
QEIs	QTN-by-environment interactions
QMDR	quantitative MDR
QQIs	QTN-by-QTN interactions
QTNs	Quantitative trait nucleotides
REMMA	rapid epistatic mixed-model association analysis
SDV	Days to flowering time under Short Day with vernalization at 4°C during 5 weeks
SNPs	single nucleotide polymorphisms
TAIR	The Arabidopsis Information Resource.
