# Supplementary figures and images for: Compressed variance component mixed model reveals epistasis associated with flowering in Arabidopsis
Source: Front Plant Sci. 2024 Jan 8;14:1283642. doi: 10.3389/fpls.2023.1283642 (PMC10800901; doi:10.3389/fpls.2023.1283642)

**A**

**LD**

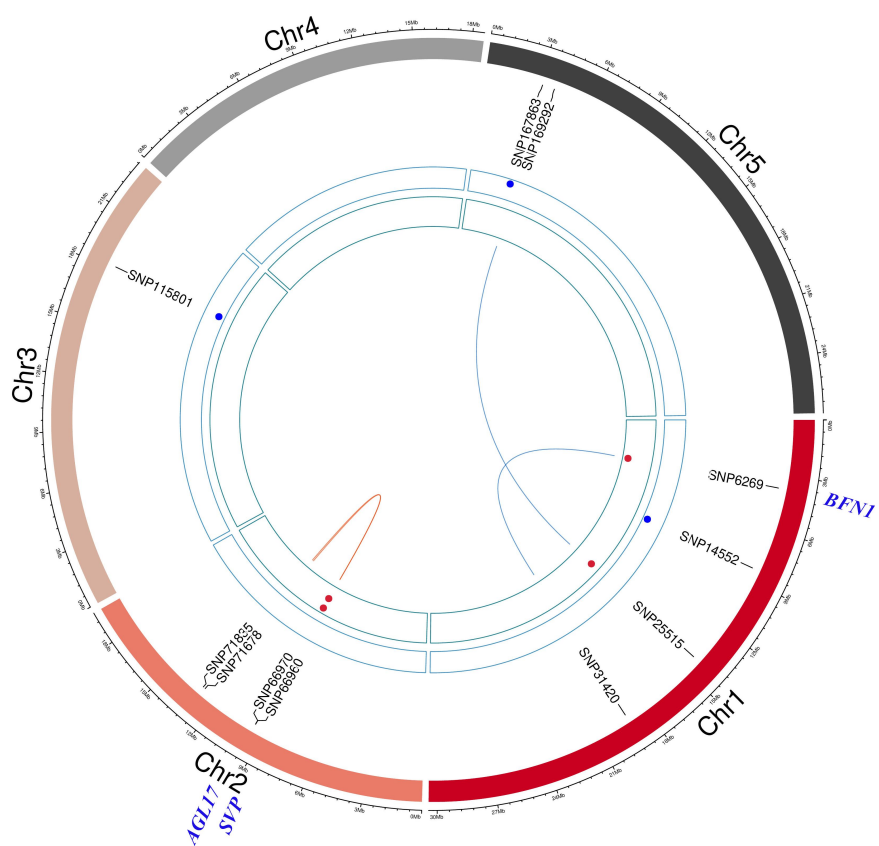

**B****SDV**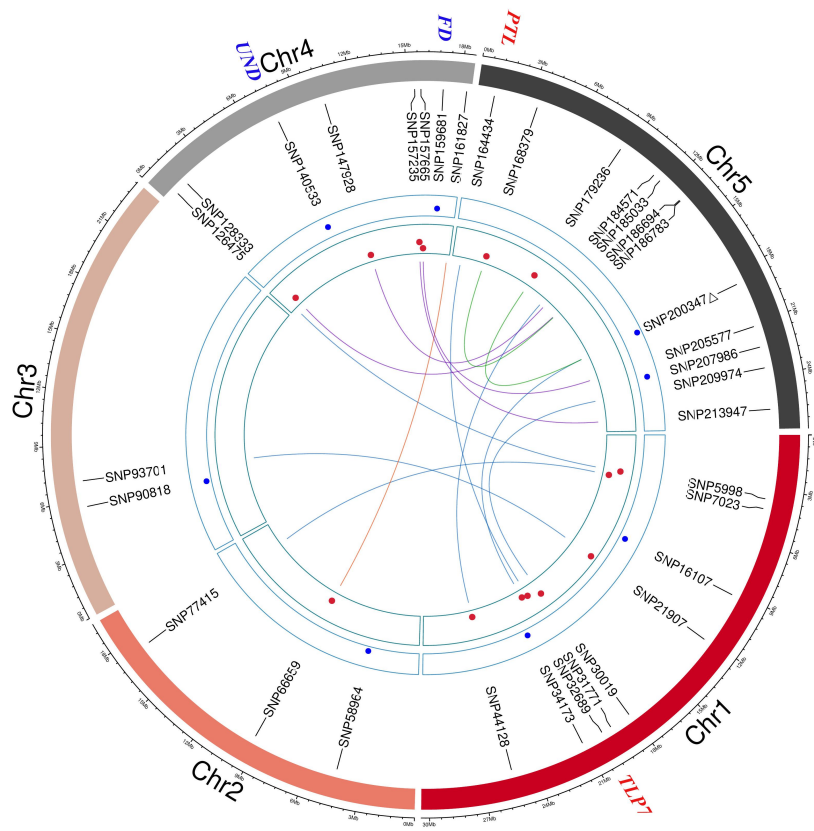

C

FT10

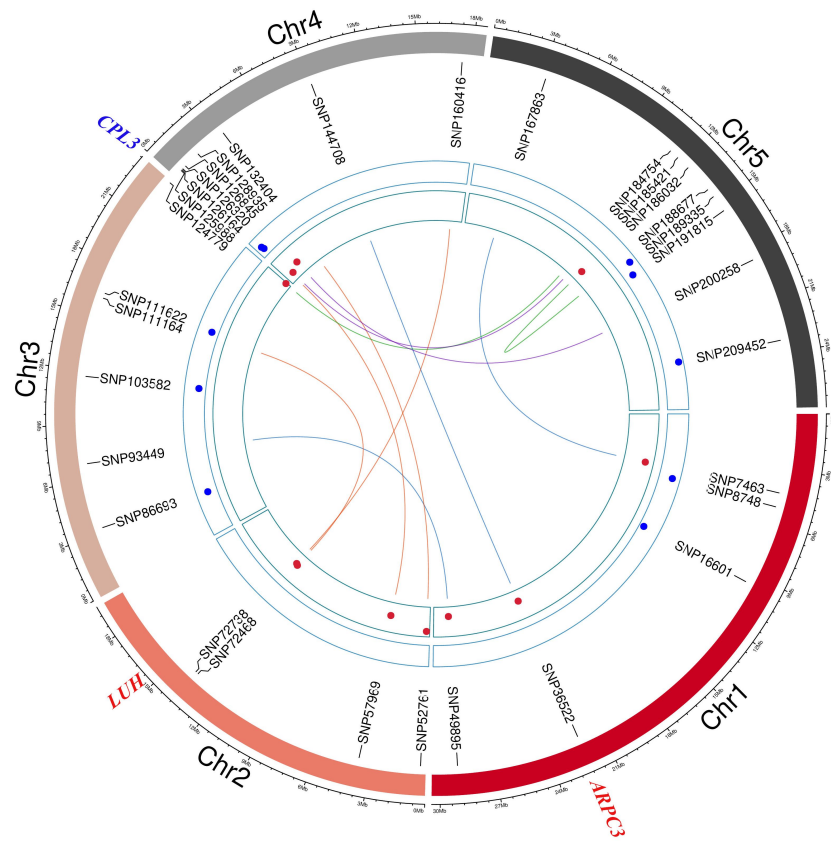

**D**

OW

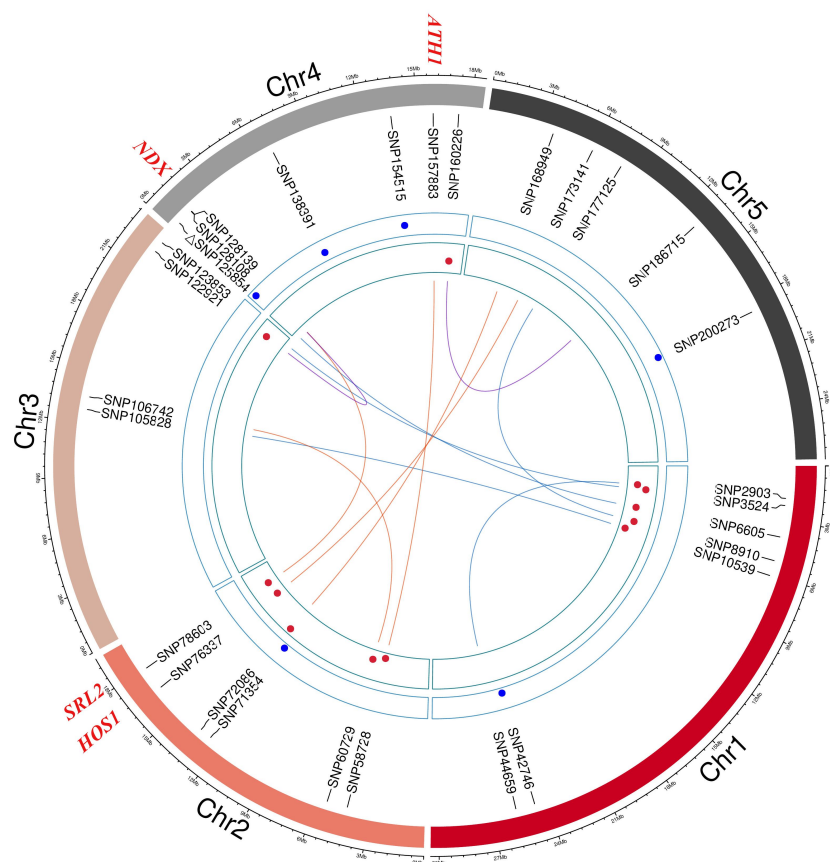

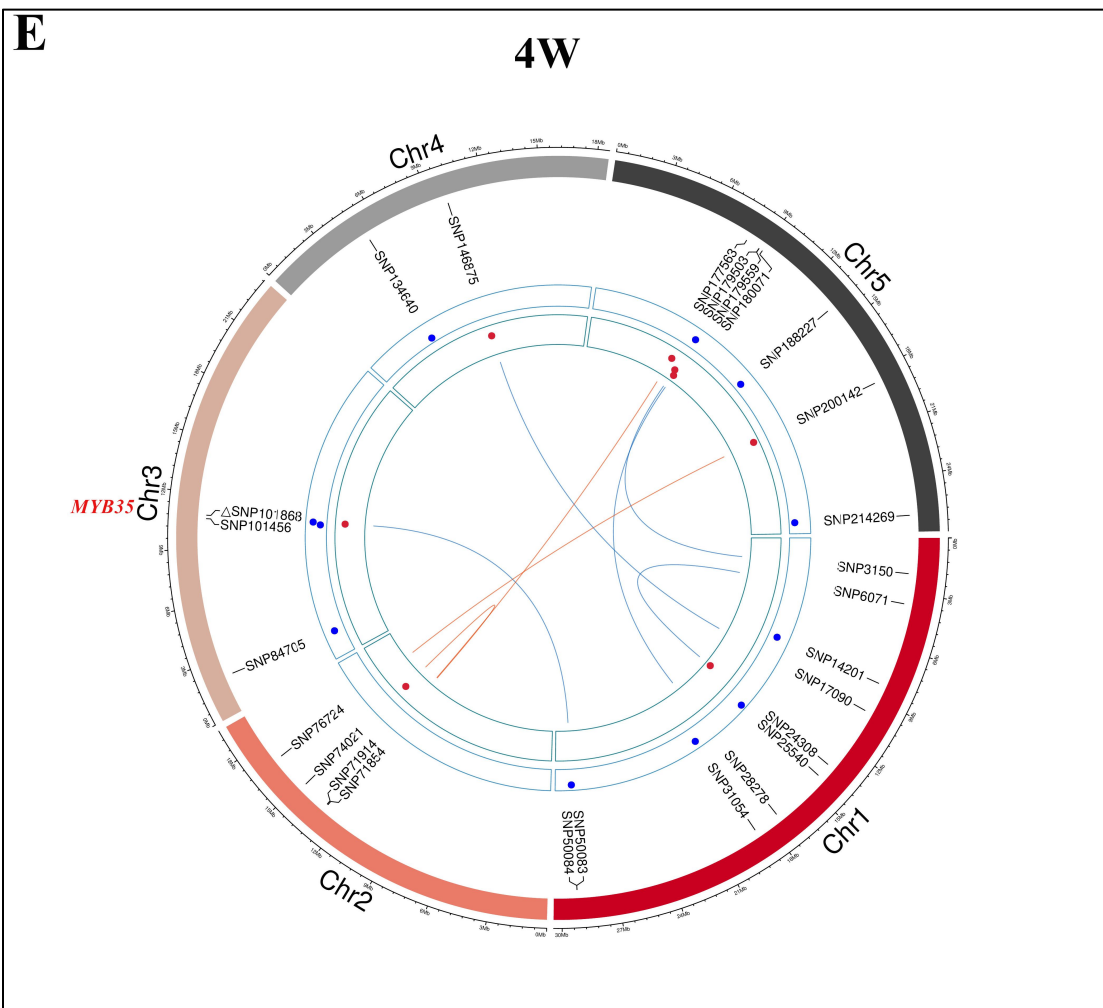

**F****8W**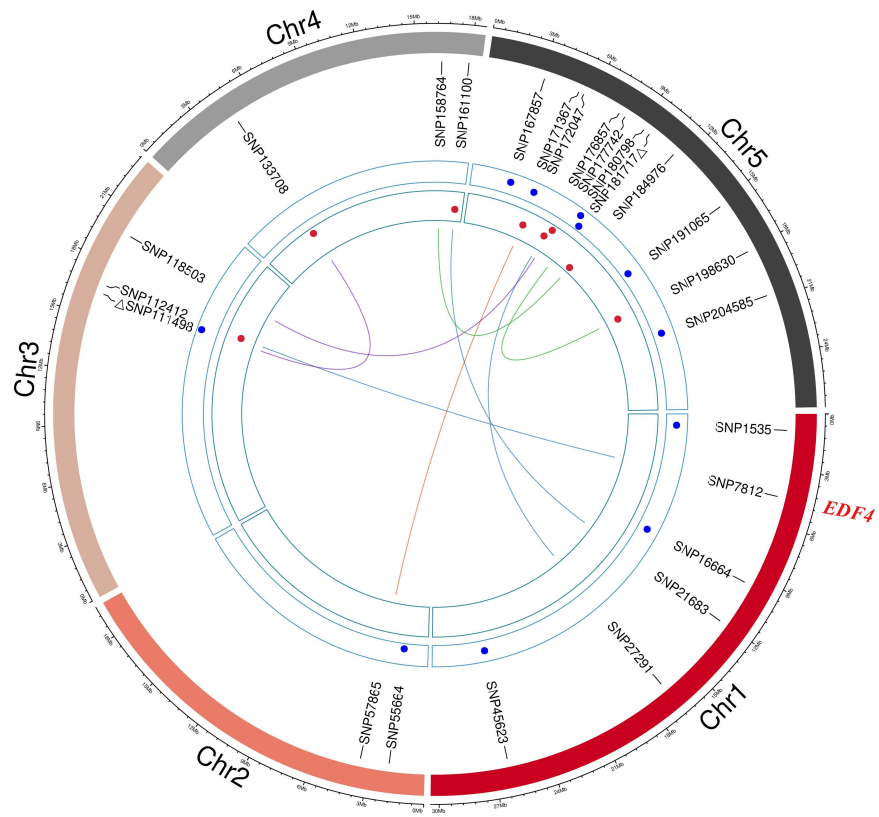

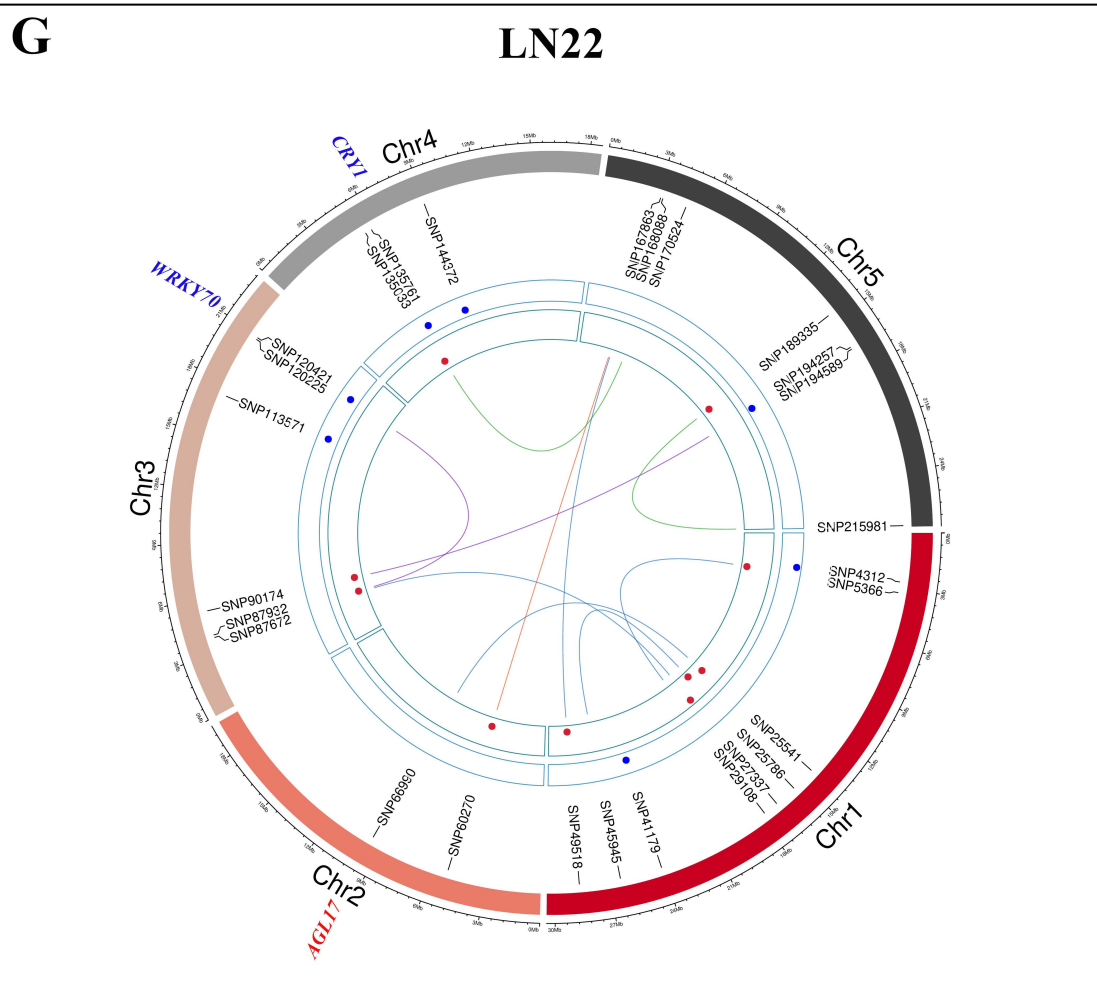

Supplement: Supplementary Figure 1 — Chord diagrams for QQIs and QTNs detected by 3VmrMLM. (A~G) correspond to the traits LD, SDV, FT10, 0W, 4W, 8W, and LN22, respectively. The inner circle displays the detected QQIs or QTNs (△ indicates overlapping loci between QQIs and QTNs), the height of red dots represents the epistatic effects of QQI pairs, and the height of blue dots represents the additive effects of corresponding QTNs. The outer circle indicates the known genes in vicinity of significant loci. [file Image_1.pdf]

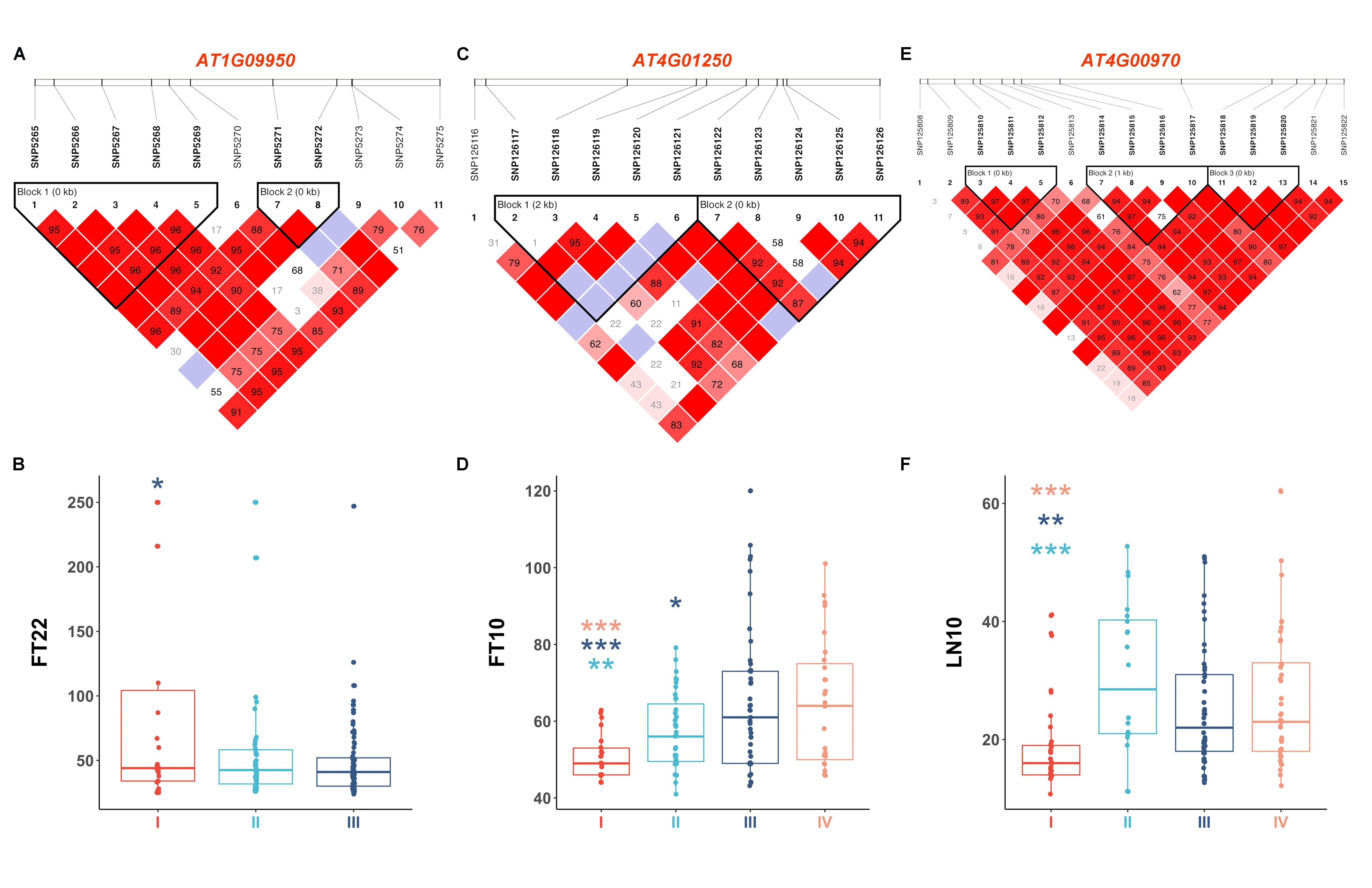

Supplement: Supplementary Figure 2 — Linkage disequilibrium and haplotype block for the candidate gene (A) AT1G09950 associated with FT22, (C) AT4G01250 associated with FT10, and (E) AT4G00970 associated with LN10. (B) Comparison of FT22 across various haplotypes I (ATATAGT), II (GAGGACT), and III (GAGGTCT). (D) Comparison of FT10 across various haplotypes I (TATACTATCT), II (TGGACCATCA), III (TGGACTAAAT), and IV (TGGACTATCT). (F) Comparison of LN10 across various haplotypes I (AGCCCACTGA), II (AGCTCGCCGT), III (CAATCGCCGT), and IV (CAATGGCCCT). For boxplots, center line shows median, box limits indicate upper and lower quartiles, and whiskers extend 1.5 times the interquartile range, while data beyond the end of the whiskers are outlying points that are plotted individually. The number of stars represents the result of t test at different significance levels (*: 0.05, **: 0.01, ***: 0.001). [file Image_2.tif]
